# A Novel Intervention for Medicaid Beneficiaries with Complex Needs

**DOI:** 10.1007/s11606-025-09839-2

**Published:** 2025-09-25

**Authors:** Luke Mueller, Neil Batlivala, Jonathan Palisoc, Connie Kim, Andrey Ostrovsky, Michael Ong, Nathan Favini

**Affiliations:** 1 Pair Team, San Francisco, CA USA; 2 Department of Health Management & Policy, School of Public Health, University of Michigan, Ann Arbor, MI USA; 3 Social Innovation Ventures, Lewes, DE USA; 4 Department of Medicine, University of California, Los Angeles, Los Angeles, CA USA; 5 School of Public Health, University of California, Berkeley, Berkeley, CA USA

**Keywords:** Medicaid, ECM, Pair Team, care management, case management, high-need, high-cost

## Abstract

**Background:**

Case management interventions may improve outcomes for patients with complex medical and social needs, though previous research has shown mixed results. Enhanced Care Management (ECM), a central element of the California Advancing and Innovating Medi-Cal (CalAIM) initiative, aims to deliver an intensive case management solution for high-needs Medi-Cal (California’s Medicaid program) members.

**Objective:**

To evaluate a novel intervention that combines ECM, telemedicine, and integrated social care delivery from community-based organizations.

**Design:**

Retrospective cohort study.

**Participants:**

Adult, ECM-eligible Medi-Cal members.

**Intervention:**

Enrollment with Pair Team, a California-based medical group and ECM provider that combines ECM, telemedicine, and integrated social care delivery from community-based organizations.

**Main Measures:**

Engagement with program and healthcare, program implementation metrics, and mental health outcomes. Pre-post analyses compared data from the year post enrollment versus year prior.

**Key Results:**

The study included 568 patients (395 [69.5%] female; average age 42.8 years). Patients averaged 3.3 program interactions per month over one year of enrollment; 17.8% of interactions were with an RN or NP. Patients engaged with Pair Team within 30 days of discharge from an ED or inpatient visit in 94.3% of visits. Post-enrollment, 300 (52.7%) patients had an HbA1c lab record (127 [22.3%] pre-enrollment, p < 0.001) and 465 (81.7%) had a blood pressure reading (423 [74.3%] pre-enrollment, p = 0.003). Post-enrollment, there was a 21% increase in outpatient visits (RR = 1.21, 95% CI 1.13–1.29), a 52% reduction in ED visits (RR = 0.48, 95% CI 0.42–0.55) and a 26% reduction in inpatient visits (RR = 0.74, 95% CI 0.55–0.99). PHQ-9 decreased by 4.0 points (p < 0.001) between intake and follow-up.

**Conclusions:**

Study participants receiving ECM services were highly engaged with the program and in their healthcare, and experienced reductions in acute care utilization and depressive symptoms. This highlights the Pair model’s potential in improving care for patients with complex needs.

**Supplementary Information:**

The online version contains supplementary material available at 10.1007/s11606-025-09839-2.

## INTRODUCTION

Over the last several decades, considerable effort has been made to improve the care of people with complex medical and social needs, sometimes referred to as “high-need, high-cost” (HNHC) patients.^1,2^ These patients often have multiple chronic conditions, behavioral health concerns, substance use disorders and social needs, like homelessness.^3^ They are known to experience higher rates of conditions like diabetes, hypertension, heart disease and cancer,^4^ and to have a high prevalence of unmet medical needs.^5^ Patients with complex medical and social needs also tend to be frequent users of high-cost services like emergency departments (ED) and inpatient facilities, with longer, higher acuity inpatient stays, and higher readmission rates.^6–8^

Numerous interventions have attempted to help HNHC patients, but results have been mixed.^1,2,9,10^ Evaluations of well-known interventions like the Camden Coalition model suggest that community-based case management may struggle to reduce acute care utilization as long as primary care and social resources are constrained.^11,12^

In this context, the state of California recently introduced the California Advancing and Innovating Medi-Cal (CalAIM) initiative; a comprehensive reform effort aimed at transforming California’s Medicaid program, Medi-Cal, to meet the needs of beneficiaries with complex medical and social needs.^13^ A crucial component of CalAIM is the Enhanced Care Management (ECM) benefit, which aims to provide focused, interdisciplinary case management for Medi-Cal enrollees who experience the greatest health disparities (i.e., preventable differences in health outcomes and access to healthcare experienced by certain populations due to demographic, economic, social and other factors).^14,15^ The benefit focuses on specific “populations of focus”, which include many patients often identified as HNHC: individuals experiencing homelessness, individuals at risk for avoidable hospital or ED utilization (also known as high-utilizers [HU]), individuals with serious mental illness (SMI), and individuals with substance use disorders (SUD).

In this manuscript, we present data on program implementation and engagement, patient engagement with healthcare (e.g., clinical testing record rates, outpatient, ED and hospital admissions visits), and behavioral health outcomes for patients in the first year of enrollment with Pair Team, a California-based medical group and provider of ECM services.

## METHODS

### Intervention

The Pair Model includes a clinical team of Lead Care Managers (LCMs), Registered Nurses (RNs), Behavioral Health Care Managers (BHCMs) who are either Licensed Clinical Social Workers or Licensed Marriage and Family Therapists, and Nurse Practitioners (NPs). LCMs come from the communities that they serve and are trained as Community Health Workers. All care team members are bilingual in English and Spanish. Patients are referred to Pair Team by a network of local Community-Based Organizations (CBOs), health system partners, and directly by Medicaid Managed Care Organizations.

The Pair Model has two notable features not required by California’s ECM program that were specifically designed to address limitations in prior programs for HNHC patients. First, to supplement limited primary care availability in the community, Pair employs a team of NPs, who care for patients via telemedicine and communicate care summaries and needs back to each patient’s primary care provider (PCP). These NPs are easily accessible and provide an additional layer of coordinated medical support to bridge care gaps between community-based PCP visits and/or ED visits. They provide post-ED and hospital discharge support, ensuring patients understand their discharge plan, have the medications they need, and have booked all recommended future appointments. They also help patients achieve their chronic disease management goals through intensive support, and can intervene before unnecessary ED visits. Pair’s NPs fax summaries of all visits to patients’ PCPs and call to speak directly to PCPs to discuss clinical information. Second, to improve access to social services, Pair has built a network of CBO partners, including homeless services organizations, shelters, and food banks. CBOs join Pair Team’s value-based care network, receive operational training and tools to better coordinate care, and are compensated for services they provide to patients. These integrations allow Pair to address social needs (e.g., housing, food and financial insecurity, transportation, social isolation, child care, etc.) quickly and in-depth with community partners that are eager to help Pair’s patients.

During intake, patients complete a detailed assessment of their health and social needs with their care team (see Appendix). LCMs form deep relationships with their patients, developing care plans, conducting motivational interviews, and becoming their trusted navigators of the health and social care system, often calling to schedule PCP visits and attending appointments with them. RNs perform patient education and triage urgent health needs; BHCMs provide behavioral healthcare services including screening, assessment, brief interventions and referral to intensive treatment; and NPs provide medical care via telemedicine focused on chronic disease management, care gap closure and avoidance of unnecessary acute care utilization. Teams are organized into pods of three LCMs, one RN, one BHCM and one NP caring for approximately 250 patients. Pods meet weekly for interdisciplinary team rounds where each patient is reviewed at enrollment, at any major change in health status (e.g., a new medical condition that requires attention, an ED visit or hospitalization, a change in social circumstance, a relapse of substance use) and at least quarterly on a routine basis.

Care teams are supported by Pair’s technology, including a custom-built case management platform (Arc) that allows for in-depth care planning and automation of clinical operations. Arc is connected to local Health Information Exchanges (HIEs) and Admission, Discharge, Transfer (ADT) systems that allow care teams to view outside health records and be alerted when patients are in the ED or hospital in order to manage transitions of care. Arc allows care team members to interact with patients via text, phone call, and email in addition to their in-person interactions.

### Participants

Participants were adult Medi-Cal members who qualified for ECM and enrolled in Pair Team’s program between July, 2022 and August, 2023. Inclusion criteria were the populations of focus defined by the State of California: individuals experiencing homelessness with at least one complex physical, behavioral, or developmental health need, individuals at risk for avoidable hospital or ED utilization (with at least five ED visits and/or three inpatient stays over six months), and individuals with SMI and SUD (who must also have one complex social factor and be high risk for institutionalization, crisis, overdose or suicide).^16^ Enrollment occurred through several channels: referrals from CBO partners, referrals from health system partners, and outreach to patients identified by Medicaid managed care plans. Children under 18 and patients without historical HIE data were excluded from the analysis. Given this study’s focus on program engagement, patients who disenrolled before one year were excluded from the primary analysis but included in a sensitivity analysis.

### Data

Demographic, enrollment, survey (including PHQ-9 forms), and Pair Team encounter data for this study was extracted from Arc. Acute and ambulatory visit data, as well as conditions, vitals, and laboratory data for this study were from HIEs, specifically the two largest national networks: CommonWell Health Alliance and Carequality. Combined they include over 75,000 provider sites and over 270 million patients across the country, including significant coverage in California. Provider sites include acute care centers, ambulatory care centers, hospitals, lab systems, pharmacies, and post-acute care centers.

### Statistical Analysis

This is a retrospective, within-group, observational cohort study. Encounters from the HIEs were classified as ED visits, inpatient visits, or outpatient visits according to the class coding of the original Consolidated-Clinical Document Architecture record. Encounters with a Pair NP or RN were not classified as outpatient visits. Any data recorded in the one year prior to enrollment (including the enrollment date) were attributed to the “pre-period”, while data in the one year after enrollment were attributed to the “post-period”.

For pre-post analyses, we assessed differences in proportions for clinical testing rates (e.g., proportion of patients with a record of a HbA1c test) and across categorical variables (e.g., PHQ-9 score categories) using the Pearson’s Chi-squared test. For differences in proportions of patients with clinical visits, we calculated p-values using a two-sided McNemar test to accommodate paired samples. Rate ratios for visits were calculated for each visit type as total post-period visits/total pre-period visits (e.g., 331/687 = 0.48). Rate ratios less than 1 thus represent a relative visit reduction across the cohort following Pair’s ECM intervention. 95% confidence intervals (CIs) for rate ratios were approximated via the Wald method in R.

### Ethical Considerations

Since the study was a secondary analysis of previously collected, de-identified data, it was deemed exempt from ethics oversight by the WCG Institutional Review Board.

## RESULTS

### Cohort

568 ECM enrollees were eligible for the study cohort [Table 1]. Over half of the study population was enrolled after being identified by Medicaid managed care plans (306, 53.9%); the rest were referred by health system partners (224, 39.4%), CBOs (17, 3.0%), or through other channels (21, 3.7%). The average age was 42.8 years and the majority self-identified as female (69.5%). 51.8% of patients were experiencing homelessness at the time of enrollment, 50.4% were experiencing SMI, 45.8% were at risk for avoidable hospital or ED utilization, and 7.6% were experiencing SUD. 45.6% of patients met criteria for multiple populations of focus. A total of 18.1% had a diabetes diagnosis and 30.5% had a hypertension diagnosis prior to enrollment. Most patients (88.0%) had not seen a PCP in the year prior to enrollment and the median time since last PCP visit was 570 days.

**Table 1:** Description of the Study Cohort

Characteristic	Overall (N = 568)
Self-reported Gender:	
Female – no. (%)	395 (69.5)
Age – no. (%)	
18–34	189 (33.3)
35–49	170 (29.9)
50–64	181 (31.9)
65 +	28 (4.9)
Age – mean (SD)	42.76 (14.33)
Race or Ethnic Group – no. (%)	
Black or African American	60 (10.6)
Hispanic or Latino	239 (42.1)
White	42 (7.4)
Other*	51 (9.0)
Unknown	176 (31.0)
Preferred Language: Spanish – no. (%)	170 (29.9)
Population of Focus – no. (%)	
HU	260 (45.8)
Homeless	294 (51.8)
SMI	286 (50.4)
SUD	43 (7.6)
Multiple PoF	259 (45.6)
Individuals with a Diabetes Diagnosis – no. (%)	103 (18.1)
Individuals with a Hypertension Diagnosis – no. (%)	173 (30.5)
2+ Chronic Conditions – no. (%)	334 (58.8)
Pre-period Health Care Visits – no. (%)	
1+ ED Visits	253 (44.5)
1+ Inpatient Visits	72 (12.7)
1+ Outpatient Visits	267 (47.0)
1+ PCP Visits	68 (12.0)
Days Since Last PCP Visit – median (IQR)	570 (168—1,387)

Legend

*Abbreviations:* ED, emergency department; Homeless, individuals experiencing homelessness; HU, individuals at risk for avoidable hospital or ED utilization; SD, standard deviation; SMI, individuals with serious mental illness; SUD, individuals with substance use disorders; PCP, primary care provider; PoF, population of focus

^*^ “Other” Race or Ethnic Group includes American Indian or Alaskan Native, Asian, Prefer not to say, and Other

### Engagement with Pair Team

**Figure 1:**
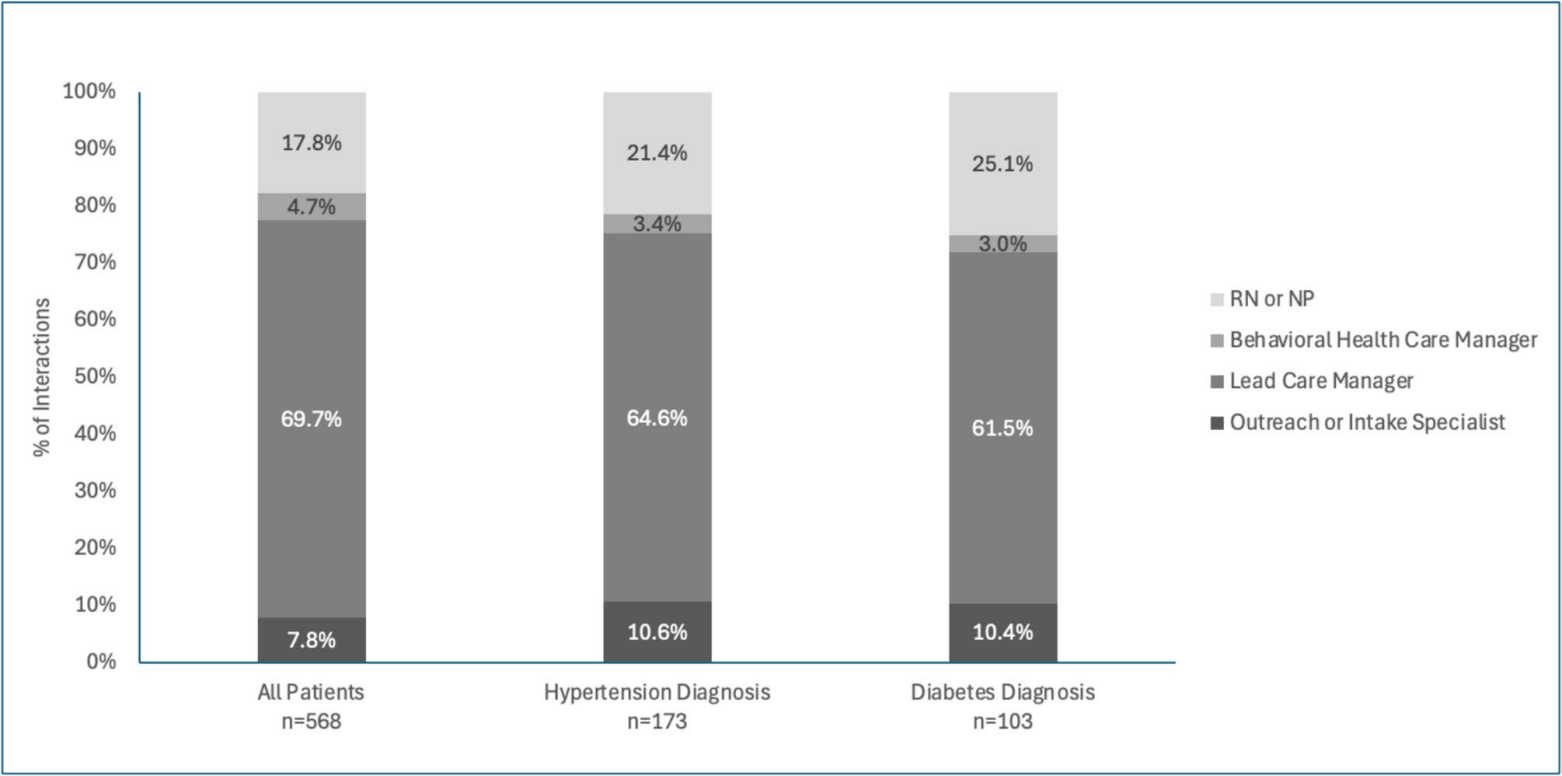
Program interactions by care team member type. Percentage of interactions with Pair’s care team in the year after program enrollment by type of care team member, for all patients as well as the cohorts with a diabetes or hypertension diagnosis. Abbreviations: *NP*, nurse practitioner; *RN*, registered nurse.

Patients had frequent interactions with Pair’s care team during the post-period. Patients averaged 3.3 (SD = 2.1) interactions per month with the care team (including in-person, text, phone call, and email interactions). Engagements were more intensive immediately after enrollment, with 4.6 engagements on average in the first month, and tapered over time to 2.4 engagements in month 12. The majority of interactions (69.7%) were with LCMs, followed by RNs or NPs (17.8%). For patients diagnosed with hypertension or diabetes, a greater percentage of interactions were with RNs or NPs [Fig. 1].

Patients also engaged with the care team after ED and inpatient discharges. A total of 407 alerts of ED or inpatient visits were received during the post-period, comprising 198 patients. After discharge, the patients in 347 (85.3%) of the visits had a follow-up encounter with the care team within 14 days of discharge, and of those, 186 (53.6%) saw a NP or RN. The patients in 384 (94.3%) of the visits had a follow-up encounter with the care team within 30 days of discharge, and of those, 232 (60.4%) saw a NP or RN.

### Engagement with Healthcare

Post-enrollment testing rates increased for patients enrolled in Pair’s program [Table 2]. In the post-period, 300 (52.7%) of all patients had an HbA1c lab record and 465 (81.7%) had a record of a blood pressure reading, compared to the pre-period where 127 (22.3%) of patients had an HbA1c lab record and 423 (74.3%) had a blood pressure reading (p < 0.001 and p = 0.003 respectively).

**Table 2:** Pre- vs. Post-enrollment Clinical Testing Rates

	Pre-period*	Post-period^†^	*P*-value^‡^
**HbA1c**			
*All Patients – no. (%)*	568 (100.0)	568 (100.0)	–
Has test – no. (%)	127 (22.3)	300 (52.7)	< 0.001
*Patients with Diabetes – no. (%)*	103 (18.1)	103 (18.1)	–
Has test – no. (%)	52 (50.5)	85 (82.5)	< 0.001
**Blood Pressure**			
*All Patients – no. (%)*	568 (100.0)	568 (100.0)	–
Has test – no. (%)	423 (74.3)	465 (81.7)	0.003
*Patients with Hypertension – no. (%)*	173 (30.5)	173 (30.5)	–
Has test – no. (%)	160 (92.5)	152 (87.9)	0.206

Legend

^*^Includes data from one year prior to enrollment

^†^Includes data one year post-enrollment

^‡^The Pearson’s Chi-squared test was used to calculate P-values

Program patients also had significant increases in outpatient visit rates following enrollment [Table 3]. Outpatient visits (excluding Pair Team encounters) increased from 1598 to 1928, representing an increase of 21% (relative risk [RR] = 1.21, 95% confidence interval [CI] 1.13–1.29) from the pre- to post-period. 47% of patients had at least 1 outpatient visit in the year prior to enrollment compared to 58% in the year post-enrollment (p < 0.001). Increases in outpatient visits were mirrored by significant reductions in acute care visits [Table 3]. Overall, ED visits decreased from 687 to 331 visits, representing a decrease of 52% (RR = 0.48, 95% CI 0.42–0.55) and inpatient visits decreased from 103 to 76 visits, representing a decrease of 26% (RR = 0.74, 95% CI 0.55–0.99) from the pre- to post-period [Table 3]. In the year prior to enrollment, 44.5% of patients had at least one ED visit, and 12.7% of patients had at least one inpatient visit, compared to 31.0% (p < 0.001) and 8.3% (p = 0.012) of patients respectively in the year post-enrollment.

**Table 3:** Pre- vs. Post-enrollment Outpatient, Emergency and Inpatient Visits

Description	Pre-period*	Post-period^†^	Rate Ratio^‡^	95% CI / *P*-value^‡^
N = 568				
**Outpatient**				
Visit count	1,598	1,928	1.21	1.13—1.29
Has 1 + visit	47.0%	57.8%	–	< 0.001
**Emergency**				
Visit count	687	331	0.48	0.42—0.55
Has 1 + visit	44.5%	31.0%	–	< 0.001
**Inpatient**				
Visit count	103	76	0.74	0.55—0.99
Has 1 + visit	12.7%	8.3%	–	0.012

Legend

*Abbreviations*: CI, confidence interval

^*^Includes data from one year prior to enrollment

^†^ Includes data one year post-enrollment

^‡^ 95% confidence intervals (CIs) for rate ratios were approximated via the Wald method. P-values for evaluating the difference in proportion of patients with visits were estimated using a two-sided McNemar test to accommodate paired samples

### Behavioral Health

There were notable changes in PHQ-9 scores observed in the post-period [Table 4]. Of the 158 individuals who had PHQ-9 data at baseline and in the post-enrollment period, 117 (74.1%) had depressive symptoms (PHQ-9 > 9) at baseline versus 57 (36.1%) with depressive symptoms in the post-enrollment period (p < 0.001). On average, patients’ total PHQ-9 scores decreased by 4.0 points (p < 0.001) between their intake and follow-up forms. The average time between the intake form and follow-up form was 240.2 days.

**Table 4:** Baseline vs. Post-enrollment PHQ-9 Scores

	Baseline	Post-period*	*P*-value^†^
PHQ-9			
Patients with baseline and post-period testing – no. (%)	158 (100.0)	158 (100.0)	–
PHQ-9 score—mean (SD)	12.7 (6.6)	8.7 (6.7)	< 0.001
PHQ-9 Severity—no. (%)			< 0.001
0–4	19 (12.0)	54 (34.2)	
5–9	22 (13.9)	47 (29.7)	
10–14	55 (34.8)	18 (11.4)	
15–19	39 (24.7)	27 (17.1)	
20 +	23 (14.6)	12 (7.6)	
Patients with depressive symptoms—no. (%)	117 (74.1)	57 (36.1)	< 0.001
Patients with suicidal ideation—no. (%)	43 (27.2)	31 (19.6)	0.144

Legend

*Abbreviations:* SD, standard deviation

^*^Average time between intake form and follow-up form was 240.2 days

^†^For continuous variables, the t-test was used to calculate P-values; for categorical variables, the Pearson’s Chi-squared test was used

### Sensitivity Analysis

The final study cohort excluded 174 patients who disenrolled from the program before one year but otherwise met inclusion criteria. We conducted sensitivity analysis including these patients (n = 742) and found that pre-post differences in clinical testing and visit rates were consistent with those for the study cohort (see Appendix). For example, outpatient visit rates increased 21%, just as in the study cohort, while ED and inpatient visits decreased 49% and 27%, compared to 52% and 26% respectively in the study cohort.

## DISCUSSION

Patients enrolled in the Pair Model engaged frequently with Pair’s care teams and received continuous, comprehensive care management, medical care and social support. They received timely follow-up care after ED visits and inpatient care, had higher HbA1c and blood pressure testing rates, and had more outpatient visits and fewer ED and inpatient visits after program enrollment. Enrollees also experienced improvement in their behavioral health, as measured by PHQ-9. The positive results of this study are an early indicator of the program’s potential benefits for patients with complex needs when coupled with accessible telemedicine and social care.

This study had several limitations. First, the study cohort was limited to patients in their first year of enrollment with Pair’s ECM program. Further longitudinal analyses are planned to understand the program’s long-term impact. Second, the study cohort does not include children under the age of 18, despite the fact that children represent a large proportion of patients enrolled with Pair Team (40%). Preliminary data suggest that interventions and utilization patterns among children are different from adults, and therefore merit separate analyses. Third, patients who disenrolled before one year were excluded from this analysis, potentially limiting the generalizability across enrolling populations. However, sensitivity analysis demonstrated that including those who disenrolled yielded similar results for pre-post differences in healthcare engagement. Finally, this is an observational study; our analysis only looks at differences within the treatment group over time and does not have a control, making it susceptible to confounding and regression to the mean.

Prior observational studies of intensive case management interventions, including the Camden Coalition model, have shown promising reductions in acute care utilization that were not observed in subsequent randomized controlled trials (RCTs), raising concerns about regression to the mean for patients with complex needs. In both the Camden RCT and a trial for high-risk patients at the VA, there were no differences in healthcare utilization between intervention and control groups.^11,17^ Evaluating the effects of the Pair Model compared to a control group is required, but there are several reasons to be hopeful about the effects observed here.

First, the Pair Model was specifically designed to address limitations identified by the RCT of the Camden Coalition model. Dr. Jeffrey Brenner, the founder of the Camden Coalition, cited “coordination to nowhere,” i.e. limited access to primary care and social resources, as the primary reason for the null result in the Camden RCT.^18^ To address this, Pair Team (1) integrated highly-accessible medical care via telemedicine into its intervention and (2) built a network of CBO partners to meet patients’ social needs. Pair’s NPs are readily available when patients have a need, for instance connecting with patients within 3 days of hospital discharge, compared to 44 days for a primary care appointment. Unlike community PCPs, NP schedules are structured so that they are available for extended visits with patients to fully address their medical needs and questions. For social needs, Pair’s LCMs navigate a network of CBOs that are eager to collaborate with Pair because the organizations are deeply partnered in a value-based care network.

Second, in contrast to many prior interventions for people with complex needs, Pair’s patients are not solely enrolled in ECM at the point of hospital discharge (which may be an inflection point in a patient’s health trajectory, past which clinical intervention is less successful),^19^ but identified through diverse channels (i.e., CBOs, providers, payers, other patients, etc.) under diverse criteria (i.e., populations of focus). Though a patient’s care plan may involve some intervention intended to mitigate ED or inpatient utilization, care plans are first and foremost developed with the patient’s long-term health in mind.

Third, Pair Team uses a novel, in-house software platform (Arc) that integrates medical and social care. This platform allows for cross-functional collaboration and coordination with third-party social service providers. HIE data is integrated into Arc, giving the team visibility into outside results (e.g. HbA1c values, BP readings) and events (e.g. hospital admissions and discharges) which automatically kick off Pair Team workflows. This assures that the Pair Model is implemented with a high degree of fidelity across all patients, addressing a key challenge identified in scaling prior interventions.^12^

Finally, this study is unique in its emphasis on the health status of HNHC patients and points toward potential benefits of the Pair Model beyond short-term reductions in healthcare utilization. The improvements in the health of HNHC patients observed here are unlikely to be the result of regression to the mean. These improvements are valuable for patients and healthcare stakeholders independent of changes in short-term acute care utilization and may portend lower rates of more serious downstream health issues and utilization over the longer term.

In summary, the Pair Model of care, which was designed to address limitations in prior interventions for patients with complex medical and social needs and delivered through California’s ECM benefit, has demonstrated promising results in the first observational study of this intervention. The high engagement rates with the program, improvements in how patients engaged with healthcare, and improvements in behavioral health outcomes observed here suggest this model has the potential to significantly improve the lives of beneficiaries, generate savings for Medicaid programs, and achieve core goals of Medicaid policymakers and health plans. Future studies should incorporate control groups in order to facilitate examination of a causal relationship between program use and outcomes and healthcare utilization.

## Supplementary Information

Below is the link to the electronic supplementary material.

Supplementary Material 1 (PDF 4.73 MB)

## Data Availability

Deidentified data related to these findings can be made available upon reasonable request.
