# Supplementary material for: A Novel Intervention for Medicaid Beneficiaries with Complex Needs
Source: J Gen Intern Med. 2025 Sep 25;41(5):1222–8. doi: 10.1007/s11606-025-09839-2 (PMC13083700; doi:10.1007/s11606-025-09839-2)
Supplement: Supplementary file 1 — Supplementary Material 1 (PDF 4.73 MB) [file 11606_2025_9839_MOESM1_ESM.pdf]

Health Assessment

Demographics

What is your first and last name?

What is your date of birth?

What is your gender?

- ◯ Female
- ◯ Male
- ◯ Non-binary
- ◯ Other

What is your sex at birth?

- ◯ Female
- ◯ Male
- ◯ Intersex

What are your preferred gender pronouns?

- ◯ He/him/his
- ◯ She/her/hers
- ◯ They/them/their
- ◯ Other

Which of the following best describes your race/ethnicity? (Select all that apply)

- ☐ American Indian or Alaska Native
- ☐ Asian

- [ ] Black or African American
- [ ] Hispanic or Latino
- [ ] Native Hawaiian or Other Pacific Islander
- [ ] White
- [ ] ASL
- [ ] Other
- [ ] Prefer not to say

What is your preferred spoken / written language?

- ◯ English
- ◯ Spanish
- ◯ Chinese
- ◯ Tagalog
- ◯ Vietnamese
- ◯ Korean
- ◯ Hindi
- ◯ Other
- ◯ Prefer not to say

If you selected 'Other' for preferred language, please specify:

How would you prefer to meet with PairTeam

- ◯ SMS/Phone
- ◯ In person

Goals

What would you like to achieve from our time together?

Social Determinants of Health

What is your current housing situation?

- ◯ Stable and safe
- ◯ Unstable
- ◯ Motel
- ◯ Staying with friends
- ◯ Temporary Shelter
- ◯ Homeless
- ◯ Other
- ◯ Declined to Answer

Are you worried about losing your housing?

- ◯ Yes
- ◯ No
- ◯ Decline to answer

Think about your living situation. Do you have any problems with any of the following? Select all that apply*

- ☐ Pests such as roaches/other bugs
- ☐ ants
- ☐ or mice/rats
- ☐ Mold
- ☐ Lead paint or pipes
- ☐ Lack of heat
- ☐ Oven or stove not working
- ☐ Smoke detectors missing or not working
- ☐ Water leaks
- ☐ None
- ☐ Declined to answer

Can you live safely and move easily around in your home?

- ◯ Yes
- ◯ No
- ◯ Not sure

- ◯ Declined to answer

In the last 12 months, how often have you been hungry or not eaten because there was not enough food in the house?

- ◯ Often
- ◯ Not often
- ◯ Not at all
- ◯ Declined to answer

How often have you run out of money to pay for food, rent, bills and medicine?

- ◯ Often
- ◯ Not often
- ◯ Not at all
- ◯ Declined to answer

What is your current work situation?

- ◯ Part-Time
- ◯ Full-Time
- ◯ Student
- ◯ Retire
- ◯ Unemployed
- ◯ Unstable (i.e. day labor)
- ◯ Other
- ◯ Prefer not to say

If you selected 'Other' for work situation, please specify:

____

In the past 12 months, has a lack of reliable transportation kept you from any of the following?

- [ ] Medical appointments
- [ ] Work
- [ ] Getting things needed for daily living
- [ ] None of the above

Does the place where you live have:

- [ ] Good lighting
- [ ] Good heating
- [ ] Good cooling
- [ ] Hot water,Indoor toilet
- [ ] Door to outside that locks
- [ ] Clear ways to exit your home
- [ ] Bed to sleep on
- [ ] Working refrigerator
- [ ] None of the above
- [ ] Declined to answer

In the past 12 months, have you been involved with the following:

- [ ] Court ordered services
- [ ] On probation
- [ ] On parole
- [ ] Re-entry program
- [ ] DUI/restricted license
- [ ] Adult Protective Services (APS)
- [ ] Child Protective Services (CPS)
- [ ] Community Legal Services
- [ ] None Prefer not to say

In the past 12 months, have you spent more than 2 nights in a row in a jail, prison, detention center, or juvenile correctional facility?

- ○ Yes
- ○ No
- ○ Declined to Answer

Are you afraid of anyone or is anyone hurting you?

- ○ Yes
- ○ No
- ○ Not sure
- ○ Declined to answer

Behavioral Health

Have you been told by a doctor or mental health provider that you have any of the following mental health conditions?

- ☐ Depression
- ☐ Bipolar Disorder
- ☐ Schizophrenia
- ☐ Anxiety
- ☐ PTSD
- ☐ Other
- ☐ None of the above

How often do you feel lonely or isolated from those around you?

- ○ Never
- ○ Rarely
- ○ Sometimes
- ○ Often
- ○ Always
- ○ Declined to answer

In the past 30 days, on how many days did you use illegal/street drugs or abuse any prescriptions?

- ○ 0
- ○ 1-3
- ○ 4-8
- ○ 9-15
- ○ 16-30
- ○ Declined to answer

In the past 30 days, on how many days did you have 4 or more alcoholic drinks (if you are female) or 5 or more alcoholic drinks (if you are male)?

- ○ 0
- ○ 1-3
- ○ 4-8
- ○ 9-15

- ◯ 16-30
- ◯ Declined to answer

In the past 30 days, how many days did you use marijuana/cannabis products?

- ◯ Never
- ◯ Seldom
- ◯ Most days
- ◯ Declined to answer

Physical Health

Have you been told by a doctor that you have any of the following medical conditions?

- ☐ Cancer
- ☐ Diabetes
- ☐ Heart Disease
- ☐ Hypertension (high blood pressure)
- ☐ Kidney disease
- ☐ Lung disease
- ☐ Stroke,None of the above

Which type of diabetes were you diagnosed with?

- ◯ Diabetes Type 1
- ◯ Diabetes Type 2
- ◯ Not sure
- ◯ Not Applicable

Are you currently pregnant?

- ◯ Yes
- ◯ No
- ◯ Declined to Answer

Have you had any of the following screenings/tests?

- [ ] Colonoscopy (in last 10 yrs)
- [ ] Stool blood test (in last 12 mo)
- [ ] Mammogram (in last 2 yrs)
- [ ] Pap smear (in last 3 yrs)
- [ ] Blood sugar (HbA1C
- [ ] in last 12 mo)
- [ ] Kidney function
- [ ] Lead screening
- [ ] None or not sure

Do you need help with any of the following actions?

- [ ] Taking your medicines
- [ ] Filling out health forms
- [ ] Answering questions during a doctor's visit
- [ ] Understanding your medical diagnosis
- [ ] Understanding child/youth member's care and treatment needs
- [ ] Filling out health forms
- [ ] Answering questions during a doctor's visit
- [ ] None of the Above

Have you given birth in the last 3 months?

- ○ Yes
- ○ No
- ○ Not Sure

Are you currently trying to become pregnant?

- ○ Yes
- ○ No
- ○ Not Sure

Are you using a form of birth control?

- ○ Birth control pills
- ○ IUD
- ○ Implant (e.g. nexplanon)
- ○ Barrier method (e.g. condoms)
- ○ Other
- ○ None

If pregnant, have you had any of the following vaccines since your pregnancy started?

- ◯ Tdap (tetanus
- ◯ diphtheria
- ◯ pertussis)
- ◯ Flu
- ◯ Not sure

Are you up to date on your vaccines?

- ◯ Yes
- ◯ No
- ◯ Not sure

When was the last time you saw your primary care provider?

- ◯ Less than 3 months ago
- ◯ Less than 6 months ago
- ◯ 6-12 months ago
- ◯ More than 1 year ago
- ◯ Not sure

How many times this year has the child/youth member received a topical fluoride treatment from a healthcare provider?

- ◯ None
- ◯ 1
- ◯ 2
- ◯ Not sure

When was the last time you had a screening or test for sexually transmitted infections?

- ◯ In the last 3 months
- ◯ In the last 6 months
- ◯ In the last year
- ◯ In the last 2 years
- ◯ Longer than 2 years

- ◯ Not sure
- ◯ Declined to answer

CHA

Do you have any problems seeing, hearing, or speaking? Select all that apply

- ☐ Seeing
- ☐ Hearing
- ☐ Speaking
- ☐ None
- ☐ Declined to answer

Are you able to reside at home with wrap around services (Ex. LTSS, DME, IHSS etc.)?

- ◯ Yes
- ◯ No
- ◯ Declined to answer

Are you, a family member, assigned caregiver, or supportive person capable of making their own decisions regarding discharge planning?

- ◯ Yes
- ◯ No
- ◯ Declined to answer

What medical equipment or supplies are you currently using? Select all that apply.

- ☐ Blood pressure monitor
- ☐ Cane
- ☐ CPAP/BPAP machine
- ☐ Diabetes supplies
- ☐ Eye glasses/Contacts
- ☐ Hearing aids
- ☐ Hospital bed/Hoyer lift
- ☐ Incontinence supplies

- [ ] Nebulizer
- [ ] Ostomy supplies
- [ ] Oxygen
- [ ] Prosthetics
- [ ] Portable toilet
- [ ] Suction supplies
- [ ] Tube feeding supplies
- [ ] Ventilator
- [ ] Walker
- [ ] Wheelchair
- [ ] Wound care supplies
- [ ] None
- [ ] Other: Free text
- [ ] Declined to answer

Do you need help obtaining any medical equipment?

- ○ Yes
- ○ No
- ○ Declined to answer

Do you need help with any of the these actions? Select all that apply.

- [ ] Taking a bath or shower
- [ ] Going up stairs
- [ ] Eating
- [ ] Getting dressed
- [ ] Brushing teeth
- [ ] brushing hair
- [ ] shaving
- [ ] Making meals or cooking
- [ ] Getting out of a bed or chair
- [ ] Shopping and getting food
- [ ] Using the toilet
- [ ] Walking
- [ ] Washing dishes or clothes
- [ ] Writing checks or keeping track of money
- [ ] Getting a ride to the doctor or to see your friends
- [ ] Doing house or yard work
- [ ] Going out to visit family or friends
- [ ] Using the phone
- [ ] Keeping track of appointments

- [ ] None
- [ ] Declined to answer

Are you getting all the help you need with these actions? **skip if you chose 'None' for the previous question. Please describe.

Do you have family members, caregiver or others willing and able to help you when you need it?

- ( ) Yes
- ( ) No
- ( ) Declined to answer

Do you ever think your family members, caregiver or others have a hard time giving you all the help you need?

- ( ) Yes
- ( ) No
- ( ) Declined to answer

Do you currently receive Care Management services through any of the following programs? Select all that apply

- [ ] CBAS
- [ ] IHSS
- [ ] AIDS Health Care Foundation Plans
- [ ] I don't know
- [ ] None
- [ ] Declined to answer
- [ ] Other

Do you qualify for IHSS?

- ( ) Yes
- ( ) No
- ( ) Declined to answer

Is it advised that you receive any Community Supports Services (Caregiver Services)

- ( ) Yes

- ◯ No
- ◯ Declined to answer

What Community Supports Services do you want or might you benefit from? **Skip this question if you answered 'no' to the previous question

- ☐ Asthma Remediation
- ☐ SNF to Home
- ☐ Environmental Accessibility Adaptations (Home Modications)
- ☐ Housing Deposits
- ☐ Housing Tenancy and Sustaining Services
- ☐ Housing Transition Navigation Services
- ☐ Medically Tailored Meals
- ☐ SNF Transition/Diversion to ALF
- ☐ Recuperative Care
- ☐ Short-Term Post-Hospitalization
- ☐ Sobering Centers

Do you have a living will or Advanced Care Directive?

- ◯ Yes
- ◯ No
- ◯ I don't know
- ◯ Declined to answer

On an average day, how would you rate your pain on a scale of 0-10, with 10 being the worst pain

[ ]

Have you had any changes in thinking, remembering, or making decisions?

- ◯ Yes
- ◯ No
- ◯ Declined to answer

Do you repeat the same things over and over (questions, stories, or statements)?

- ◯ Yes
- ◯ No
- ◯ Declined to answer

Do you have trouble learning how to use a tool, appliance, or gadget?

- ◯ Yes
- ◯ No
- ◯ I don't know
- ◯ Declined to answer

Do you forget the correct month or year?

- ◯ Yes
- ◯ No
- ◯ I don't know
- ◯ Declined to answer

Do you have trouble handling complicated financial affairs (paying bills, taxes)?

- ◯ Yes
- ◯ No
- ◯ I don't know
- ◯ Declined to answer

Do you have trouble remembering appointments?

- ◯ Yes
- ◯ No
- ◯ I don't know
- ◯ Declined to answer

Have you fallen in the last month?

- ◯ Yes
- ◯ No

Are you afraid of falling?

- ◯ Yes
- ◯ No
- ◯ Declined to answer

Is anyone using your money without your OK?

- ◯ Yes
- ◯ No
- ◯ Declined to answer

In general, how would you rate your health?

- ◯ Excellent
- ◯ Very good
- ◯ Good
- ◯ Fair
- ◯ Poor
- ◯ Declined to answer

What are one or two of your top health and wellness goals?

What are your challenges in achieving your health and wellness goals?

What is your current height in inches?

What is your current weight in pounds?

Do you participate in any physical activity such as walking or biking?

- ◯ Yes
- ◯ No
- ◯ Declined to answer

Have you been seen by any of the following types of doctors? Select all that apply.

- ☐ Addiction specialist
- ☐ Allergist
- ☐ Behavioral health

- [ ] Cardiologist
- [ ] Dermatologist
- [ ] Endocrinologist
- [ ] Gastroenterologist
- [ ] Hematologist
- [ ] Nephrologist
- [ ] Neurologist
- [ ] OBGYN
- [ ] Oncologist
- [ ] Orthopedist
- [ ] Pain Management
- [ ] Palliative Care
- [ ] Podiatrist
- [ ] Pulmonologist
- [ ] Urologist
- [ ] None
- [ ] I don't know
- [ ] Declined to answer

When did you last see the dentist?

Do you have any current issues with your teeth?

- ◯ Yes
- ◯ No
- ◯ I don't know
- ◯ Declined to answer

Do you need assistance in obtaining referrals to any specialists?

- ◯ Yes
- ◯ No
- ◯ I don't know
- ◯ Declined to answer

In the past 12 months, how many times have you been admitted to a hospital? (admitted to the hospital or psychiatric facility for at least one night)

- ◯ 0

- ◯ 1
- ◯ 2+
- ◯ Declined to answer

Reason for admission

[ ]

In the past 3 months, how many times have you gone to the emergency room for care?

- ◯ 0
- ◯ 1
- ◯ 2
- ◯ 3+
- ◯ Declined to answer

Reason for admission

[ ]

In the past 30 days, have you stayed overnight in a skilled nursing home?

- ◯ Yes
- ◯ No
- ◯ I don't know
- ◯ Declined to answer

Are you taking all your prescribed medications?

- ◯ Yes
- ◯ No
- ◯ I don't know
- ◯ Declined to answer

Are you a part of a spiritual community, or do you consider yourself spiritual or religious?

- ◯ Yes
- ◯ No

- I don't know
- Declined to answer

Does your spirituality inuence your health care decision making?

- Yes
- No
- Declined to answer

Do you have cultural beliefs that affect how you receive treatment or care?

- Yes
- No
- Declined to answer

In an event of an emergency (natural disaster, power outage, no-show for support person), do you have a plan of action?

- Yes
- No
- Declined to answer

Do you use tobacco or nicotine products?

- Yes
- No
- Declined to answer

Rate your readiness for quitting tobacco or nicotine on a scale of 1-10. (1 means not interested, and 10 means extremely interested) *Skip if you don't use tobacco or nicotine products

In the past 30 days, how many days did you have 4 or more (females) or 5 or more (males) alcoholic drinks?

- 0
- 1-3
- 4-8
- 9-15

- ◯ 16-30
- ◯ Declined to answer

Submit Assessment

Supplemental Text

Cohort Details

568 adult patients were included in the final study cohort, however, there were 1,022 total patients enrolled with Pair Team between July, 2022 and August, 2023. 280 children under the age of 18 at enrollment (27.3% of cohort) were excluded from the analysis given their many differences in eligibility, intervention, and outcome patterns compared to adults. Future research is intended for these patients. In addition, 174 adult patients (23.5% of the remaining cohort) were excluded from the cohort due to early disenrollment (i.e., disenrollment less than a year after enrollment). The most common reason for early disenrollment was the patient having no contact with the care team for at least three months. Others disenrolled because of meeting their care goals or moving geographies or to a new health plan or health system. Disenrolled patients were enrolled with Pair Team for an average of 239.0 days (standard deviation [SD] = 74.9 days).

To examine if there were differences between those who disenrolled (23.5%) and those in the study cohort who remained enrolled for at least one year (76.5%), we compared baseline characteristics between the cohorts. Demographic characteristics were similar between disenrolled patients and study cohort patients, with no observable differences in self-reported gender, age, or geography. There were however, notable differences in population of focus (PoF), with disenrolled patients more likely to be experiencing homelessness (62.1% of disenrolled cohort vs. 51.8% of study cohort, p=0.029), and less likely to be at risk for avoidable hospital or ED utilization (29.9% of disenrolled

cohort vs. 45.8% of study cohort, p<0.001). There was no significant difference between cohorts for SMI-, SUD-, or multiple-eligible PoF patients. Fewer disenrolled patients had ED visits in the year prior to enrollment compared to patients in the study cohort (31.0% of disenrolled compared to 44.5% of study cohort), but there was no observable difference in inpatient, outpatient, or PCP visit rates in the year prior to enrollment.

We also conducted sensitivity analysis on healthcare engagement metrics to determine if study results differed when the disenrolled cohort was included. Results remained consistent with those for the study cohort. In the post-period, 362 (48.8%) of patients had an HbA1c lab testing record (170 [22.9%] pre-enrollment, p<0.001) and 594 (80.1%) had a record of a blood pressure reading (541 [72.9%] pre-enrollment, p=0.001). Outpatient visits increased from 1906 to 2302 visits, representing an increase of 21% (relative risk [RR]=1.21, 95% confidence interval [CI] 1.14-1.28) from the pre- to post-period. 45.8% of patients had at least 1 outpatient visit in the year prior to enrollment compared to 55.3% in the year after enrollment (p<0.001). ED visits decreased from 786 to 404 visits, representing a decrease of 49% (RR=0.51, 95% CI 0.46-0.58) and inpatient visits decreased from 125 to 91 visits, representing a decrease of 27% (RR=0.73, 95% CI 0.56-0.95) from the pre- to post-period. In the year prior to enrollment, 41.4% of patients had at least one ED visit, and 11.6% of patients had at least one inpatient visit, compared to 29.4% (p<0.001) and 7.5% (p=0.006) of patients respectively in the year after enrollment

HIE Data

Health Information Exchange (HIE) data represents a patient's clinical history typically formatted as Consolidated-Clinical Document Architecture (CCDA) documents, which in turn can be translated into FHIR resources. HIEs operate on local as well as national levels, and in this study, we relied on data from the two largest national networks: CommonWell Health Alliance and Carequality, which combined include more than 75,000 provider sites, and over 270 million patients.

An advantage of HIE data over the more commonly-used administrative claims, or electronic health record (EHR) datasets, is that it does not require imposing continuous eligibility criteria; patients can move between providers, plans, or lines of business and remain within the dataset. In the study cohort, some patients had records dating back decades. Nevertheless, to ensure network coverage, patients were required to have at least one encounter (i.e., ED visit, inpatient stay, or outpatient visit) in the pre- or post-enrollment period.

To prepare the data for statistical analysis, we partnered with Zus Health for further processing, enrichment, and validation of the data. Zus provides extensive documentation on its platform [link], but for this study, the most notable components were the following: 1) translation of FHIR resources to a relational database; 2) de-duplication of records reported by multiple entities; and 3) terminology normalization.

Defining chronic conditions

To identify patients with multiple chronic conditions, we leveraged Zus's terminology normalization and mappings to standard ontologies. In particular, chronic conditions were identified by the Chronic Condition Indicator (CCI) ontology, while distinct

conditions were identified by the Clinical Classifications Software Refined (CCSR) ontology. As an example, the two of the most common chronic conditions by CCI and CCSR categories in the study cohort were Essential Hypertension and Diabetes Mellitus with Complication; if a patient had both of these conditions during the pre-period, they were included in the 2+ chronic conditions subgroup.
